# Supplementary material for: Loss of the Mitochondrial Fission GTPase Drp1 Contributes to Neurodegeneration in a Drosophila Model of Hereditary Spastic Paraplegia
Source: Brain Sci. 2020 Sep 17;10(9):646. doi: 10.3390/brainsci10090646 (PMC7564485; doi:10.3390/brainsci10090646)
Supplement: Supplementary file 1 [file brainsci-10-00646-s001.pdf]

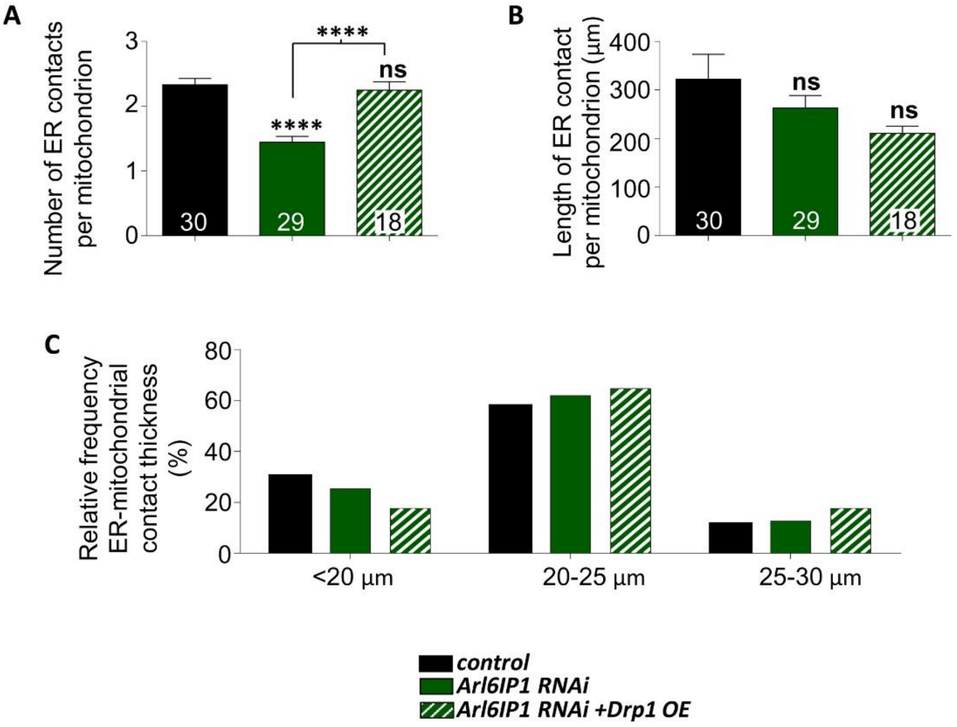

Figure S1. Analysis of ER-mitochondrial contacts in *Arl6IP1* knockdown *Drosophila*. Further analysis of electro micrographs of larvae as described in Figure 2. Graphs show quantification of number of ER contacts per mitochondrion (**A**), total length of ER-mitochondrial contacts per mitochondrion (**B**) and frequency distribution of the thickness of ER-mitochondrial contacts (**E**) in the three genotypes studied. Data are expressed as means ± SEM (*n* = 18-30 cells from three independent experiments) and values significantly different from control were determined by one-way ANOVA and Tukey's multiple comparisons test (****, $P < 0.0001$; ns, $P > 0.05$).

Table S1. Statistical comparison of climbing assays of male flies generated by crossing *nSyb-GAL4* flies to w^{1118} (control), UAS-*Arl6IP1* RNAi (*Arl6IP1* RNAi), UAS-*Arl6IP1* RNAi.*UAS-Drp1*^{WT} (*Arl6IP1* RNAi + *Drp1*^{WT}) or UAS-*Arl6IP1* RNAi.*UAS-Drp1*^{K38A} (*Arl6IP1* RNAi + *Drp1*^{K38A}).

Tukey's multiple comparisons test	*P* value summary
**Day 3**	
control vs. *Arl6IP1* RNAi (GD)	ns
control vs. *Arl6IP1* RNAi (GD) + Drp1^{WT}	ns
control vs. *Arl6IP1* RNAi (GD) + Drp1^{K38A}	*
Arl6IP1 RNAi (GD) vs. *Arl6IP1* RNAi (GD) + Drp1^{WT}	ns
Arl6IP1 RNAi (GD) vs. *Arl6IP1* RNAi (GD) + Drp1^{K38A}	ns
Arl6IP1 RNAi (GD) + Drp1^{WT} vs *Arl6IP1* RNAi (GD) + Drp1^{K38A}	ns
**Day 9**	
control vs. *Arl6IP1* RNAi (GD)	ns
control vs. *Arl6IP1* RNAi (GD) + Drp1^{WT}	ns
control vs. *Arl6IP1* RNAi (GD) + Drp1^{K38A}	****
Arl6IP1 RNAi (GD) vs. *Arl6IP1* RNAi (GD) + Drp1^{WT}	ns
Arl6IP1 RNAi (GD) vs. *Arl6IP1* RNAi (GD) + Drp1^{K38A}	****
Arl6IP1 RNAi (GD) + Drp1^{WT} vs *Arl6IP1* RNAi (GD) + Drp1^{K38A}	****

**Day 18**	
control vs. *Arl6IP1* RNAi (GD)	***
control vs. *Arl6IP1* RNAi (GD) + $Drp1^{WT}$	ns
control vs. *Arl6IP1* RNAi (GD) + $Drp1^{K38A}$	****
Arl6IP1 RNAi (GD) vs. *Arl6IP1* RNAi (GD) + $Drp1^{WT}$	*
Arl6IP1 RNAi (GD) vs. *Arl6IP1* RNAi (GD) + $Drp1^{K38A}$	**
Arl6IP1 RNAi (GD) + $Drp1^{WT}$ vs *Arl6IP1* RNAi (GD) + $Drp1^{K38A}$	****
**Day 24**	
control vs. *Arl6IP1* RNAi (GD)	****
control vs. *Arl6IP1* RNAi (GD) + $Drp1^{WT}$	ns
control vs. *Arl6IP1* RNAi (GD) + $Drp1^{K38A}$	****
Arl6IP1 RNAi (GD) vs. *Arl6IP1* RNAi (GD) + $Drp1^{WT}$	***
Arl6IP1 RNAi (GD) vs. *Arl6IP1* RNAi (GD) + $Drp1^{K38A}$	****
Arl6IP1 RNAi (GD) + $Drp1^{WT}$ vs *Arl6IP1* RNAi (GD) + $Drp1^{K38A}$	****
**Day 30**	
control vs. *Arl6IP1* RNAi (GD)	****
control vs. *Arl6IP1* RNAi (GD) + $Drp1^{WT}$	*
control vs. *Arl6IP1* RNAi (GD) + $Drp1^{K38A}$	****
Arl6IP1 RNAi (GD) vs. *Arl6IP1* RNAi (GD) + $Drp1^{WT}$	ns
Arl6IP1 RNAi (GD) vs. *Arl6IP1* RNAi (GD) + $Drp1^{K38A}$	****
Arl6IP1 RNAi (GD) + $Drp1^{WT}$ vs *Arl6IP1* RNAi (GD) + $Drp1^{K38A}$	****
**Day 36**	
control vs. *Arl6IP1* RNAi (GD)	****
control vs. *Arl6IP1* RNAi (GD) + $Drp1^{WT}$	**
control vs. *Arl6IP1* RNAi (GD) + $Drp1^{K38A}$	****
Arl6IP1 RNAi (GD) vs. *Arl6IP1* RNAi (GD) + $Drp1^{WT}$	ns
Arl6IP1 RNAi (GD) vs. *Arl6IP1* RNAi (GD) + $Drp1^{K38A}$	****
Arl6IP1 RNAi (GD) + $Drp1^{WT}$ vs *Arl6IP1* RNAi (GD) + $Drp1^{K38A}$	****

Table S2. Statistical comparison of climbing assays of male flies generated by crossing *nSyb-GAL4* flies to w^{1118} (control), UAS-*Arl6IP1* RNAi (*Arl6IP1* RNAi), UAS-*Arl6IP1* RNAi.*UAS-*$Drp1^{WT}$ (*Arl6IP1* RNAi + $Drp1^{WT}$) or UAS-*Arl6IP1* RNAi.*UAS-*$Drp1^{K38A}$ (*Arl6IP1* RNAi + $Drp1^{K38A}$).

Tukey's multiple comparisons test	*P* value summary
**Day 3**	
control vs. *Arl6IP1* RNAi (KK)	ns
control vs. *Arl6IP1* RNAi (KK) + $Drp1^{WT}$	ns
control vs. *Arl6IP1* RNAi (KK) + $Drp1^{K38A}$	ns
Arl6IP1 RNAi (KK) vs. *Arl6IP1* RNAi (KK) + $Drp1^{WT}$	ns
Arl6IP1 RNAi (KK) vs. *Arl6IP1* RNAi (KK) + $Drp1^{K38A}$	ns
Arl6IP1 RNAi (KK) + $Drp1^{WT}$ vs *Arl6IP1* RNAi (KK) + $Drp1^{K38A}$	ns
**Day 9**	
control vs. *Arl6IP1* RNAi (KK)	ns
control vs. *Arl6IP1* RNAi (KK) + $Drp1^{WT}$	ns
control vs. *Arl6IP1* RNAi (KK) + $Drp1^{K38A}$	*

Arl6IP1 RNAi (KK) vs. *Arl6IP1* RNAi (KK) + $Drp1^{WT}$	ns
Arl6IP1 RNAi (KK) vs. *Arl6IP1* RNAi (KK) + $Drp1^{K38A}$	ns
Arl6IP1 RNAi (KK) + $Drp1^{WT}$ vs *Arl6IP1* RNAi (KK) + $Drp1^{K38A}$	*
**Day 18**	
control vs. *Arl6IP1* RNAi (KK)	*
control vs. *Arl6IP1* RNAi (KK) + $Drp1^{WT}$	ns
control vs. *Arl6IP1* RNAi (KK) + $Drp1^{K38A}$	***
Arl6IP1 RNAi (KK) vs. *Arl6IP1* RNAi (KK) + $Drp1^{WT}$	*
Arl6IP1 RNAi (KK) vs. *Arl6IP1* RNAi (KK) + $Drp1^{K38A}$	ns
Arl6IP1 RNAi (KK) + $Drp1^{WT}$ vs *Arl6IP1* RNAi (KK) + $Drp1^{K38A}$	***
**Day 24**	
control vs. *Arl6IP1* RNAi (KK)	**
control vs. *Arl6IP1* RNAi (KK) + $Drp1^{WT}$	ns
control vs. *Arl6IP1* RNAi (KK) + $Drp1^{K38A}$	****
Arl6IP1 RNAi (KK) vs. *Arl6IP1* RNAi (KK) + $Drp1^{WT}$	*
Arl6IP1 RNAi (KK) vs. *Arl6IP1* RNAi (KK) + $Drp1^{K38A}$	ns
Arl6IP1 RNAi (KK) + $Drp1^{WT}$ vs *Arl6IP1* RNAi (KK) + $Drp1^{K38A}$	****
**Day 30**	
control vs. *Arl6IP1* RNAi (KK)	*
control vs. *Arl6IP1* RNAi (KK) + $Drp1^{WT}$	ns
control vs. *Arl6IP1* RNAi (KK) + $Drp1^{K38A}$	****
Arl6IP1 RNAi (KK) vs. *Arl6IP1* RNAi (KK) + $Drp1^{WT}$	ns
Arl6IP1 RNAi (KK) vs. *Arl6IP1* RNAi (KK) + $Drp1^{K38A}$	*
Arl6IP1 RNAi (KK) + $Drp1^{WT}$ vs *Arl6IP1* RNAi (KK) + $Drp1^{K38A}$	****
**Day 36**	
control vs. *Arl6IP1* RNAi (KK)	ns
control vs. *Arl6IP1* RNAi (KK) + $Drp1^{WT}$	ns
control vs. *Arl6IP1* RNAi (KK) + $Drp1^{K38A}$	****
Arl6IP1 RNAi (KK) vs. *Arl6IP1* RNAi (KK) + $Drp1^{WT}$	ns
Arl6IP1 RNAi (KK) vs. *Arl6IP1* RNAi (KK) + $Drp1^{K38A}$	****
Arl6IP1 RNAi (KK) + $Drp1^{WT}$ vs *Arl6IP1* RNAi (KK) + $Drp1^{K38A}$	****

Table S3. *n* numbers and median lifespan for survival assays of male flies generated as in Tables S1 and S2.

**Genotype**	***n* number**	**median life span**
control (GD)	61	54
Arl6IP1 RNAi (GD)	59	56
Arl6IP1 RNAi (GD) + $Drp1^{WT}$	63	56
Arl6IP1 RNAi (GD) + $Drp1^{K38A}$	57	54
control (KK)	76	49
Arl6IP1 RNAi (KK)	74	65
Arl6IP1 RNAi (KK) + $Drp1^{WT}$	74	56
Arl6IP1 RNAi (KK) + $Drp1^{K38A}$	68	55.5

Table S4. Statistical comparison of survival assays of male flies generated as in Tables S1 and S2.

**Log-Rank (Mantel Cox) test**	***P* value**	***P* value summary**
control (GD) vs *Arl6IP1* RNAi (GD)	0.5556	ns
control (GD) vs *Arl6IP1* RNAi (GD) + $Drp1^{WT}$	0.4496	ns
control (GD) vs *Arl6IP1* RNAi (GD) + $Drp1^{K38A}$	0.6898	ns
control (KK) vs *Arl6IP1* RNAi (KK)	<0.0001	****
control (KK) vs *Arl6IP1* RNAi (KK) + $Drp1^{WT}$	0.266	ns
control (KK) vs *Arl6IP1* RNAi (KK) + $Drp1^{K38A}$	0.0096	**
